# A cryo-EM grid preparation device for time-resolved structural studies

**DOI:** 10.1107/S2052252519011345

**Published:** 2019-09-05

**Authors:** Dimitrios Kontziampasis, David P. Klebl, Matthew G. Iadanza, Charlotte A. Scarff, Florian Kopf, Frank Sobott, Diana C. F. Monteiro, Martin Trebbin, Stephen P. Muench, Howard D. White

**Affiliations:** a School of Biomedical Sciences, University of Leeds, Leeds LS2 9JT, UK; b Astbury Centre for Structural and Molecular Biology, University of Leeds, Leeds LS2 9JT, UK; c School of Molecular and Cellular Biology, University of Leeds, Leeds LS2 9JT, UK; d The Hamburg Centre for Ultrafast Imaging, Universität Hamburg, Hamburg, Germany; e Department of Chemistry, Biomolecular and Analytical Mass Spectrometry group, University of Antwerp, Antwerp, Belgium; f Department of Chemistry,State University of New York at Buffalo, New York, USA; g Department of Physiological Sciences, Eastern Virginia Medical School, Norfolk, Virginia USA

**Keywords:** time-resolved cryo-EM, sample preparation, microscope hardware, structural biology, voltage-assisted spraying

## Abstract

Structural biology generally provides static snapshots of protein conformations that can provide information on the functional mechanisms of biological systems. Time-resolved structural biology provides a means to visualize, at near-atomic resolution, the dynamic conformational changes that macromolecules undergo as they function. X-ray free-electron-laser technology has provided a powerful tool to study enzyme mechanisms at atomic resolution, typically in the femtosecond to picosecond timeframe. Complementary to this, recent advances in the resolution obtainable by electron microscopy and the broad range of samples that can be studied make it ideally suited to time-resolved approaches in the microsecond to millisecond timeframe to study large loop and domain motions in biomolecules. Here we describe a cryo-EM grid preparation device that permits rapid mixing, voltage-assisted spraying and vitrification of samples. It is shown that the device produces grids of sufficient ice quality to enable data collection from single grids that results in a sub-4 Å reconstruction. Rapid mixing can be achieved by blot-and-spray or mix-and-spray approaches with a delay of ∼10 ms, providing greater temporal resolution than previously reported mix-and-spray approaches.

## Abstract 2

Time-resolved structural studies are becoming an important tool in understanding biological function. Here we describe a cryo-EM grid freezing device capable of rapidly mixing and plunge freezing grids within 10 ms.

## 1. Introduction   

Our fundamental understanding of biological processes is often underpinned by structural biology, which ultimately may assist our ability to design tailored medicines through structure-based drug design (Blundell, 2017 ▸). Advances in technology are allowing us to determine the structure of more complicated and challenging systems. Yet, these methods typically yield structural snapshots of single states of otherwise dynamic protein systems. Better understanding of molecular mechanisms in biology requires time resolution, which is restricted by current technology. X-ray free-electron-laser technology can resolve side-chain movements during catalysis on the picosecond to minute timescale, but its use is limited to samples that form ordered micro-crystals (Suga *et al.*, 2017 ▸; Stagno *et al.*, 2017 ▸). Cryo-electron-microscopy (cryo-EM) is not constrained by the crystal lattice and allows for structure determination of large non-symmetric macromolecular complex structures in solution, to near-atomic resolution (Smith & Rubinstein, 2014 ▸). Cryo-EM has been used to visualize large structural changes in proteins and protein complexes on the millisecond timescale (Frank, 2017 ▸) and used to determine the structure of multiple conformational states exhibited by numerous macromolecular systems, such as the ribosome and rotary F-ATPase (Fernández *et al.*, 2013 ▸; Zhou *et al.*, 2015 ▸). However, it has proven difficult to trap different kinetic sub-states and, although computational sorting can provide different conformations, it does not provide information on the order of catalysis, or the lifetime of the intermediates (Nakane *et al.*, 2018 ▸).

The recent renaissance in cryo-EM has been fuelled by rapid developments in microscopes, direct electron detectors and ever more sophisticated data-processing algorithms, but a current bottleneck still resides within the sample-preparation procedure, which has changed little over the last 30 years. There have been recent advances in grid-production technology such as Spotiton and VitroJet, which show great promise in producing high-quality and consistent ice but they are not currently capable of time-resolved applications (Dandey *et al.*, 2018 ▸; Ravelli *et al.*, 2019 ▸).

Time-resolved cryo-EM (TrEM) is an ideal method to study conformational changes occurring on the microsecond to millisecond timescale (Subramaniam & Henderson, 1999 ▸; Kaledhonkar *et al.*, 2018 ▸), providing snapshots of conformational states at sub-nanometre resolutions that can be put in order to understand mechanisms on a broad range of specimens. TrEM experiments have previously been demonstrated using a blot-and-spray approach; applying the protein of interest to an EM grid and pre-blotting before spraying substrate to initiate a reaction, followed by rapid plunging into liquid ethane to vitrify and stop the reaction after a specific time delay. This was demonstrated by the ground-breaking work on the acetyl­choline receptor (Unwin, 1995 ▸). Through spraying of substrate mixed with a fiducial marker (ferritin) upon a pre-blotted EM grid, and taking account of the diffusion edge of the substrate, it was possible to achieve time-resolutions as low as 2 ms for the mixing of acetyl­choline with the acetyl­choline receptor (Unwin & Fujiyoshi, 2012 ▸). This approach can be challenging because of difficulties in getting consistent mixing across an EM grid, and requires high substrate concentrations and fiducial markers. An alternative is the mix-and-spray approach where substrate and protein can be pre-mixed before directly spraying onto a fast-moving EM grid, which is plunged into liquid ethane (Feng *et al.*, 2017 ▸). This technique has shown considerable success for studies of ribosome mechanics (Kaledhonkar *et al.*, 2019 ▸).

Here we report a significantly improved device which provides greater reproducibility and more detailed characterization than previous designs. This is the first apparatus that permits both blotting and spraying of EM grids, as well as rapid mixing, and that takes advantage of the benefits of voltage-assisted spraying (typically 5 kV). The resulting grids are of sufficient quality for sub-4 Å resolution structure determination and allow rapid mixing and freezing in the millisecond time frames (>10 ms). These capabilities will allow us to determine different conformational states of protein complexes with a time resolution not accessible through conventional EM-grid preparation methods.

## 2. Materials and methods   

### 2.1. Sample preparation   

Apoferritin from equine spleen was obtained from Sigma–Aldrich (A3660), and dialysed into the target buffer {20 m*M* HEPES [4-(2-hydroxyethyl)-1-piperazineethanesulfonic acid], 150 m*M* NaCl pH 7}. *Escherichia coli* ribosomes were prepared in 50 m*M* HEPES pH 7.5, 100 m*M* KAc (potassium acetate) and 8 m*M* MgAc_2_ (magnesium acetate). Porcine ventricular thin filaments were prepared as previously described (Siemankowski & White, 1984 ▸) and diluted to 10 µ*M* in 10 m*M* MOPS [3-(*N*-morpholino)propanesulfonic acid] pH 7, 50 m*M* KAc, 3 m*M* MgCl_2_ and 1 m*M* EGTA prior to use. G-actin used for the blot/spray experiment was polymerized according to a protocol by Professor Peter Knight (personal communication). For the blotting/spraying experiment, 30 µ*M* apoferritin (24-mer) was sprayed onto a grid which was pre-blotted with 2 µ*M* F-actin (3 µl, 4 s blot time). For the mixing/spraying experiment with apoferritin and thin filaments, 30 µ*M* apoferritin and 10 µ*M* thin filaments were used and mixed in a 1:1 ratio. For the actomyosin dissociation experiment, a solution of 20 µ*M* thin filaments with 24 µ*M* myosin Va(1IQ)S1 in 10 m*M* MOPS pH 7, 50 m*M* KAc, 3 m*M* MgCl_2_ and 1 m*M* EGTA was rapidly mixed (1:1) with buffer containing 0, 0.016, 0.16 or 1.6 m*M* ATP (making final ATP concentrations of 0, 8, 80 and 800 µ*M*, respectively).

### 2.2. Grid preparation   

Before spraying/plunge freezing grids, all syringe pumps were initialized and the generated spray was examined visually. Compressed N_2_ gas was bubbled through water to increase humidity in the spraying chamber to avoid possible evaporation of the spray in flight. The position of the sprayer was adjusted, using a trial grid to ensure it was aligned. A Styrofoam system from a Mark IV Vitrobot was used to provide a reservoir of liquid nitro­gen and to house the liquid ethane. Valves controlling plunger speed and N_2_ flow were adjusted to provide the desired flow rate through the nozzle and the desired plunger speed. The tubing and syringes of the plunge freezing apparatus was washed with 3 × 50 µl ddH_2_O (purified water), followed by 3 × 50 µl of the respective buffer and loaded with 33 µl protein solution (the dead volume between the valves and the sprayer is 25–35 µl). Cryo-EM grids (Quantifoil R2/1 Cu mesh 200–300 or Quantifoil R1.2/1.3 Cu mesh 300) were glow discharged with air plasma in a Cressington 208 carbon coater with a glow-discharge unit for 90 s (10 mA/0.1 mbar air pressure) and used within 30 min to avoid hydro­philic recovery of the grids. For grid preparation, negative-pressure tweezers holding a grid were mounted into the plunging arm of the instrument, and the environmental chamber of the instrument was closed allowing the humidity to reach ≥80–90%. Then, 33 µl of protein solution was loaded into a 100 µl glass syringe (Kloehn). The ethane cup was placed in the target position and the high voltage was turned on. A software­-controlled system was used to control the flow rates and timing. Typically, the solution was sprayed at 8.3 µl s^−1^ for 3.5 s (to stabilize the spray) before plunging through the spray. A pressure of 0.5–2.5 bar was applied to the piston causing the grid to accelerate and reach the spray in less than 0.1 s (distance: 4 cm). The grid passed through the spray, accelerated further over the remaining 3 cm to the ethane surface and terminated by a mechanical stop leaving the grid ∼1 cm below the surface of the liquid ethane.

The grid was then transferred into liquid N_2_ and stored until screening. For the mix/spray technique, two syringes were used, one with each reactant, and the flow rate was reduced accordingly to 4.2 µl s^−1^ for each syringe, resulting in the same overall flow rate. For the blot-and-spray technique, 3 µl of the first protein sample was placed onto the grid, which was subsequently blotted with two strips of Whatman 43 filter paper for 4 s. After blotting, the blotter was retracted and the grid plunged through the spray (which had been initiated during the blotting step to ensure stabilization) into the liquid ethane. Upon completion of all the experiments the syringes and tubing were washed with buffer and water.

### 2.3. Data collection and processing   

All cryo-EM imaging was done on an FEI Titan Krios microscope equipped with a Falcon III direct electron detector operating in integrating mode (Astbury BioStructure Laboratory). The main data acquisition and processing parameters are listed in Table S1 in the Supporting information. All processing was done using *RELION*2.1 (Kimanius *et al.*, 2016 ▸) and *RELION*3 beta (Zivanov *et al.*, 2018 ▸). For all three datasets, the processing was done as follows: micrographs were motion corrected with the *RELION*3 implementation of *MotionCor2* (Zheng *et al.*, 2017 ▸) and then the contrast transfer function (CTF) for each micrograph was estimated using *Gctf* (Zhang, 2016 ▸). For apoferritin and ribosome, references for automated particle picking were generated from class averages obtained from a small number of manually selected particles. The results of the template-based automated picking were manually inspected. Then, 2D classification was used to select particles with high-resolution information, which were taken forward to generate an initial 3D reconstruction. After two rounds of CTF refinement and Bayesian particle polishing the final reconstruction was obtained. The presented structures were filtered by local resolution.

For the ribosome dataset, 3D classification with six classes was used to sort out 50S subunits reducing the particle number from 47 866 to 34 010. For the thin-filament dataset, the *RELION* tools for helical processing were employed (He & Scheres, 2017 ▸). All filaments were manually picked. As an initial model, either a 60 Å low-pass filtered structure of actin (PDB entry 5mvy; Paul *et al.*, 2017 ▸) or a featureless cylinder were used. Both gave nearly identical results with the low-pass filtered PDB model leading to slightly higher resolution. CTF refinement was not found to be beneficial in this case. The final reconstruction was subjected to 3D classification with eight classes to extract a tropomyosin-containing structure (9 496 particles) which after 3D refinement had a resolution of 10.4 Å. Helical symmetry for both structures was applied using the refined values for twist and rise with the *relion_helix_toolbox* program. Fourier shell correlation was determined by the two half-map gold-standard method for which curves are shown in Fig. S1 in the Supporting information.

### 2.4. Mixer design   

The mixer/sprayer was constructed from standard high-performance liquid chromatography fittings and tubing, and the air tip from a 10 µl Gilson pipette tip (Fig. S2). The spray tip [Fig. S2(*a*)] was constructed from a 2–3 mm length of 150/40 µm polyimide-coated quartz tubing (Molex) glued into 360/180 µm tubing with polyacrylate glue. The 360 tubing was sealed into 1/16′′ outer diameter (OD) fluorinated ethylene propylene (FEP) tubing (Upchurch) with a 0.015′′ inner diameter (ID). The spray tip was positioned 0–0.5 mm past the end of the air tip. Connection to the high voltage was via a short piece of 0.007′′ platinum wire used to make contact with the solution via a ‘T’ connector with 1/16′′ OD, 0.01′′ ID FEP tubing. The voltage of the high-voltage supply could be varied from 2 to 10 kV by varying the input from a low-voltage DC power supply (Celex BPS1510) to an EMCO Q101N-5 high-voltage converter. The high voltage was measured from the voltage across the 100 KΩ section of a 100 MΩ–100 KΩ voltage-divider circuit using a digital voltmeter (Radio Shack). The valves and pistons of the syringe pumps and forceps holding the EM grid were all grounded to prevent possible damage from stray high voltage.

For the mixing units [Fig. S2(*b*)], two concentric tubes (360/200 µm and 165/100 µm) (Molex) were used so that mixing of the two solutions would not occur until just prior to spraying. FEP tubing (0.007′′ ID 1/16′′ OD) was used to seal the inner 165 µm capillary. The interior tubing was positioned 100–200 µm from the beginning of the spray tip to minimize the dead time. The mixer’s geometry approximates that of a double back-to-back ‘T’ mixer.

## 3. Results   

### 3.1. Optimization of the setup   

**Figure 1:**
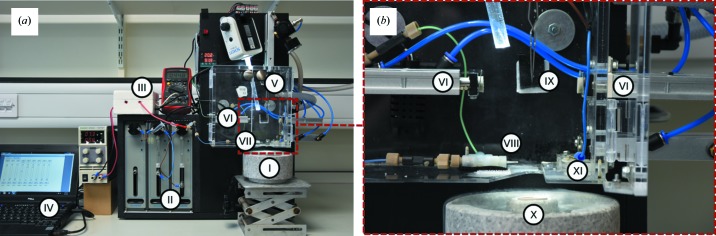
Current setup of the TrEM apparatus. (*a*) Overview of the complete apparatus showing the Styrofoam freezing cup which houses the liquid ethane (I), syringe pumps (II), high-tension voltage module (III), computer controller (IV), forceps on plunger (V), blotting arms (VI) and sprayer (VII). (*b*) Zoomed-in view of the spray chamber showing the spray nozzle (VIII), blotting arms (VI), forceps with grid (IX), ethane cup within the Styrofoam liquid nitro­gen holder (X), and a port that opens just prior to the grid plunge and limits the exposure of the liquid ethane to the humid air in the chamber (XI).

The modified setup described in this work (Fig. 1 ▸) is based on a previous design (White *et al.*, 2003 ▸), to which a number of significant adaptations have been applied, a humidity chamber has been added and the sprayer design optimized to provide efficient mixing and droplet sizes. These improvements have been made possible through better characterization of droplet speed and size, and the ability to collect Atlas low magnification views of each grid to better assess ice thickness and consistency. To improve spray distribution and produce a fine aerosol of droplets, a 5 kV potential is used for voltage-assisted spraying, which has significant advantages over standard spraying using only air pressure. With this approach, the behaviour of the spray can be more stringently controlled so as to obtain optimal ice thickness whilst requiring less sample. Droplets produced by voltage-assisted spraying are highly charged, promoting their self-dispersion and preventing coalescence (Jadhav *et al.*, 2011 ▸). Additionally, a humidity-controlled chamber is used to provide reproducible conditions and ensure that the micro-droplet spray does not evaporate en-route to the EM grid. The system is operated through computer-controlled syringe drivers (Kloehn 50300 series) that control the timing of and flow of the spraying, and additionally provide software control of sample blotting and plunging. The basic design has been described in detail in a previous review (Kaledhonkar *et al.*, 2018 ▸).

As in the case of traditional blotting, plasma treatment of the grid’s surface is an essential step in reducing the inherent hydro­phobicity of the carbon coated grids; otherwise very few, if any, droplets adhere to the grid. In addition to the time of mixing, the plunge speed of the grid was used to alter the delay time and time resolution of the experiments. In our apparatus, we use dual chamber air pistons (Fig. 1 ▸) to blot the grids (VI), accelerate the grid into the liquid ethane (I), and open and close the port opening (XI). To better define the parameters and speeds at which grids could be plunged, a range of air pressures (0.5–2.5 bar) were used, producing plunge velocities between 1 and 3 m s^−1^ (see Movie S1 in the Supporting information).

**Figure 2:**
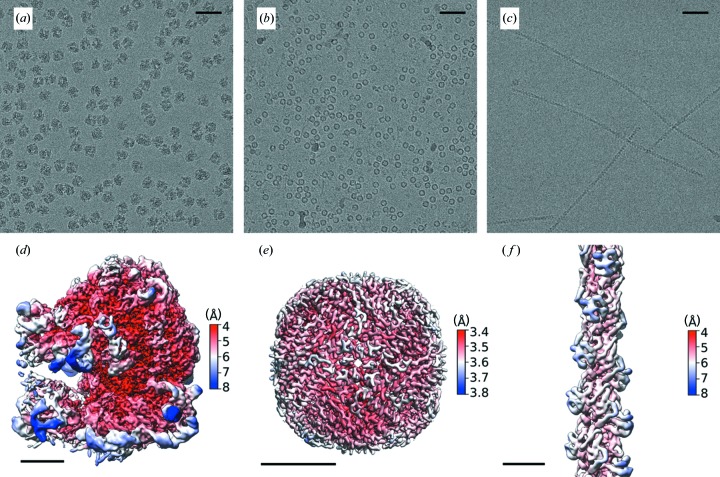
Structures of the three model systems from grids prepared on the TrEM setup. To test our ability to make high-quality EM grids by spraying proteins on a fast-moving plunging grid, three samples were tested and data are shown with representative micrographs and 3D reconstructions: (*a*, *d*) *E. coli* ribosome (0.72 µ*M* in 50 m*M* HEPES pH 7.5, 100 m*M* KAc, 8 m*M* MgAc_2_), (*b*, *e*) apoferritin [30 µ*M* (24-mer) in 20 m*M* HEPES pH 7.5, 150 m*M* NaCl], and (*c*, *f*) porcine thin filaments [5 µ*M* (actin monomer) in 10 m*M* MOPS pH 7, 50 m*M* KAc, 3 m*M* MgCl_2_, 1 m*M* EGTA]. The scale bars in (*a*), (*b*) and (*c*) represents 50 nm. The scale bars in (*d*), (*e*) and (*f*) represents 5 nm.

We initially investigated the ability of the TrEM set-up to produce grids with ice of sufficient quality and to collect cryo-EM data capable of generating high-resolution structures. Three different model systems (apoferritin, ribosome, and thin filaments) were used to investigate the ability to spray protein samples onto a fast-moving glow-discharged EM grid. Data for each system were collected from single grids, using a Falcon 3 detector running in integrating mode on an overnight run (∼12 h). From overview images, we observed ∼20–50% of the grids’ surface area covered with droplets. Although many droplets produced ice too thick for data collection, the grids showed many areas of ice that were suitable for imaging and subsequent high-resolution structure determination (Fig. S3). For each sample, this resulted in ∼1 800 holes with suitable ice for imaging with the ability to take multiple exposures per hole permitting a maximum of 6 000 micrographs per grid (see Table S2). For apoferritin, a total of 1 772 micrographs were collected, with the resulting reconstruction producing a global resolution of 3.6 Å [Figs. 2 ▸(*a*) and 2 ▸(*b*)]. For the *E. coli* ribosome, 1 494 micrographs were collected, with 34 010 particles contributing to a final reconstruction that had a global resolution of 4.3 Å [Figs. 2 ▸(*c*) and 2 ▸(*d*)] and showed a wide sampling of angular orientations [Fig. S1(*c*)]. Porcine cardiac thin filaments were studied to investigate the applicability of the apparatus on long thin filamentous particles. The resulting grids showed good distribution of the specimen within the holes, with no signs of damage in terms of filament length [Fig. 2 ▸(*c*)]. The resulting reconstruction of the actin core of the thin filaments was resolved to 5.6 Å with the entire thin filament having a lower resolution [10.4 Å, Fig. S1(*b*)] because of the heterogeneity of the attached tropomyosin. Of the micrographs collected a significant number went into the final reconstruction, demonstrating the consistency in the ice with ∼86%, 97% and 100% of the collected micrographs containing particles that went into the final reconstruction of the apoferritin, ribosome and F-actin, respectively [Fig. S4(*a*)]. Tomographic analysis of the ice shows that it varies between 80 and 125 nm, which is thicker than ideal and limits the resolution of the data. Moreover, Thon ring analysis of all collected micrographs shows the main peak lies between 3 and 5 Å [Fig. S4(*b*)]. Since the resolution of the apoferritin dataset is not particle-number limited (>800 000 asymmetric units), ice thickness is the main determinant of resolution and we conclude that thinner ice will be required to obtain >3.5 Å. However, it should be noted that the ability to rapidly mix and spray even at resolutions <3.5 Å can provide new insights into protein mechanism and cycling.

### 3.2. Estimation of plunging and droplet speed   

**Figure 3:**
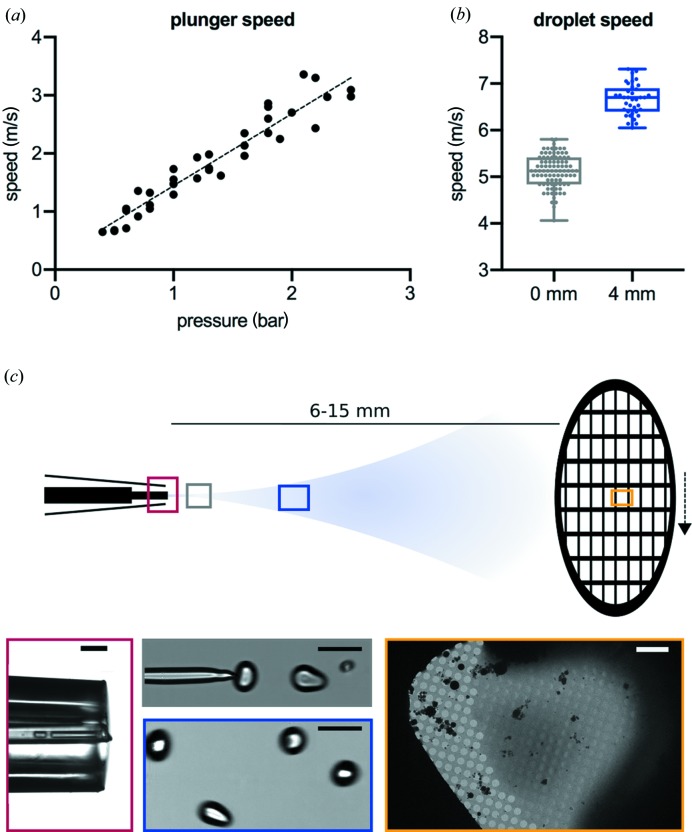
Measuring plunger and droplet speeds. (*a*) The relationship between pressure and plunger speed, linear fit: speed (m s^−1^) = ∼1.5 × pressure (bar). (*b*) A boxplot of droplet speeds with ten different droplets tracked over at least three frames for each position, measured at the spray tip (0 mm) and at a distance of 4 mm. (*c*) Microscopic images of the spray tip (red, scale bar 200 µm), the breakup point of the liquid jet (grey, scale bar 100 µm), 4 mm away from the capillary tip (blue, scale bar 100 µm) and a grid square of a vitrified grid in the electron microscope (yellow, scale bar 10 µm).

In a mix-and-spray type of experiment, the total amount of time that the mixed substrates interact can be calculated if we know the speed of the plunger arm, the distance travelled and the speed of the droplets after leaving the mixer/sprayer. The speed of the pneumatic plunging arm was determined using a linear potentiometer. Although, it should be noted that the plunger continuously accelerates as the grid moves towards the ethane container but reaches near maximal velocity at the point of the spray. Therefore, the measurement is a single measure of velocity at the position of the spraying nozzle and it will only slightly underestimate the overall plunging speed. With a constant distance of 3 cm between the central point of the spray cone and the ethane surface, we calculate a time between 30 and 10 ms (for a pressure range of 0.5–2.5 bar) for the grid to reach the ethane once the sample has been applied [Fig. 3 ▸(*a*)]. Droplet speeds were calculated based on high-speed video recordings of sprayed droplets at the tip of the nozzle, as well as at a distance of 4 mm from the nozzle [green/blue square in Fig. 3 ▸(*b*)]. Both yield droplet speeds of greater than 4 m s^−1^ [Fig. 3 ▸(*c*)]. We also observed acceleration of the droplets as they moved away from the sprayer. With a tip/grid distance of 6–15 mm, this equates to a time of flight of ≤4 ms for the slowest droplets and the longest distance. Higher plunge speeds result in fewer droplets adhering to the grid, with the shortest possible delay on the current setup equating to ∼11 ms from the time the droplet leaves the spray nozzle to it being vitrified on the grid (with a typical drop speed of 6 m s^−1^, a sprayer-to-grid distance of 6 mm and a spray-to-ethane distance of 3 cm). Analysis of the droplets on the high-speed videos shows an approximate diameter of 75 µm [Fig. 3 ▸(*c*)]. This is considerably larger than previously reported (1 µm), which used electrospray (White *et al.*, 2003 ▸) rather than voltage-assisted spraying, but produces ice suitable for high-resolution data collection.

**Figure 4:**
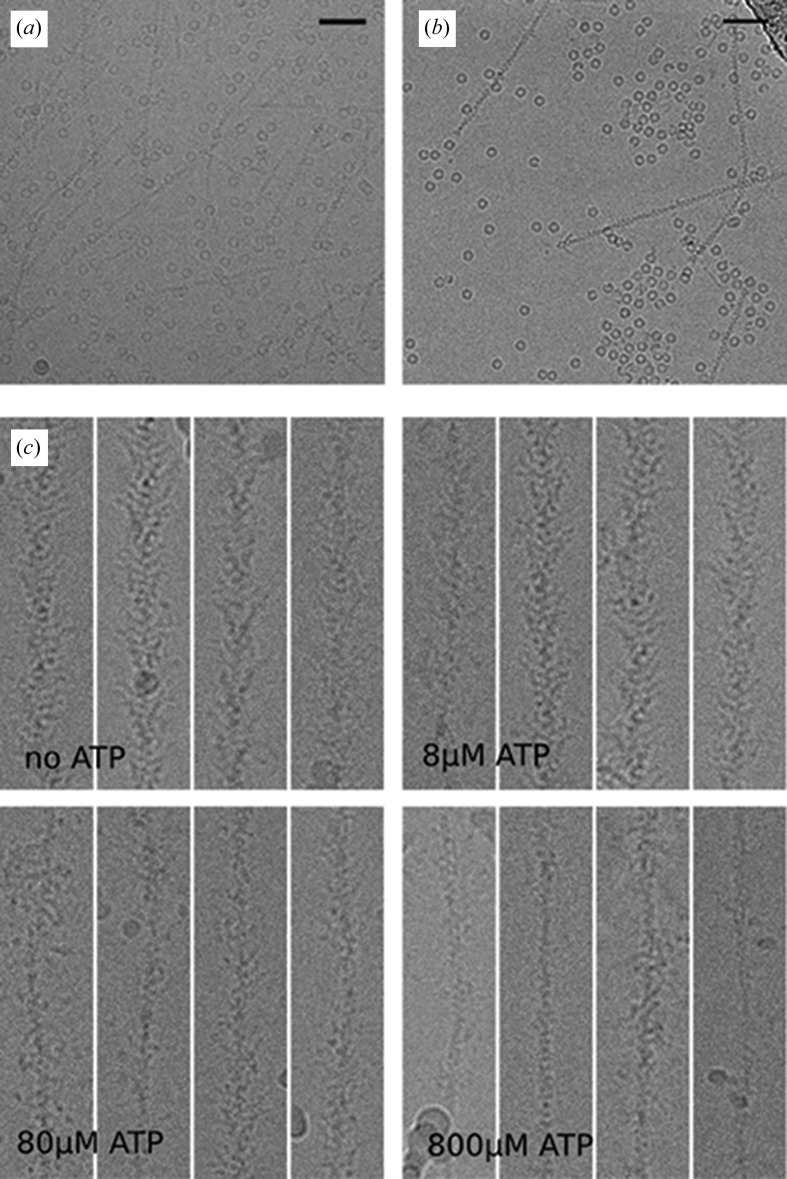
Rapid mixing of samples on the TrEM setup. (*a*) Representative micrograph with thin filaments blotted and apoferritin sprayed on the subsequent plunged grid. (*b*) Representative micrograph from the rapid mixing of apoferritin and thin filaments, and direct spraying onto the EM grid. (*c*) Four representative images of myosin S1 decorated filaments after the rapid mixing (∼15 ms) of 0, 8, 80 and 800 µ*M* MgATP showing decoration consistent with that predicted by kinetic modelling (0, 10, 70 and 99%, respectively).

In order to examine the applicability of the new apparatus for the preparation of time-resolved samples, we validated the mixing capability using several different approaches. The first was to conventionally blot the grid containing the first protein and subsequently pass the blotted grid through a voltage-assisted spray containing the second protein (Movie S2). By removing the dead time associated with the mixing chamber and the in-flight time of the droplets, the time delay between protein deposition on the grid and freezing is ∼10 ms, and is dependent only on the distance between the sprayer and the liquid ethane (3 cm), and the plunge speed (≤3 m s^−1^). Actin filaments were pre-blotted on the grid (blot time 4 s) and then passed through a voltage-assisted spray of apoferritin. Good quality and clear co-localization could be detected for the samples [Fig. 4 ▸(*a*)]. The second approach was to mix apo­ferritin and thin filaments within the capillary tube just prior to voltage-assisted spraying with the resulting ice showing clear mixing of both samples in all areas studied [Movie S3 and Fig. 4 ▸(*b*)]. The added delay time, resulting from the additional volume prior to spraying is 1–2 ms, and the time of flight from the spray trip to the grid was measured to be less than 4 ms resulting in an overall time delay of ∼15 ms. Although both of these approaches provided promising results and showed clear co-localization, the next step was to provide clear evidence of mixing. To achieve this, we rapidly mixed and vitrified two samples by mixing actomyosin-S1 with varying concentrations of MgATP, which dissociates the myosin-S1 from the actin with a second-order rate constant of 10^6^ *M* ^−1^ s^−1^ (Siemankowski & White, 1984 ▸). The extent of dissociation of S1 from actin after ∼15 ms of mixing of 0, 8, 80 and 800 µ*M* MgATP is predicted to be 0, 10, 70 and >99%, respectively. By maintaining the plunger speed in a way that the TrEM setup was working with an ∼15 ms delay, the resulting filament decoration seen in the microscope was consistent with that expected, demonstrating that the two solutions were mixed for ∼15 ms, prior to freezing [Fig. 4 ▸(*c*)]. Investigations are currently ongoing to determine the degree of mixing within the chamber, with provisional modelling suggesting most of the mixing may occur within the droplets in-flight. With the increased time of mixing and time of flight for the sample, the approximate resulting time resolution of this approach is in the range of 10–15 ms. Using refined microfluidic mixers, it may be possible to achieve more complete mixing before spraying the mixed sample.

## 4. Discussion   

Electron microscopy has seen significant developments over the last ten years with numerous high-resolution structures of previously intractable protein systems. However, despite the first reported TrEM experiment being conducted in 1994 by the pioneering work of Nigel Unwin, progress has been slow (Berriman & Unwin, 1994 ▸). With the recent developments in cryo-EM hardware and software, developing a reliable TrEM setup is becoming a reality. In this work, we reported a system which can produce cryo-EM grids with a minimum time delay between mixing and freezing of 10 ms, faster than the previously reported fastest speed of 24 ms for a mix-and-spray device (Fu *et al.*, 2019 ▸). Through three model systems we have shown the capability of producing high-quality EM data at a resolution sufficient for resolving side-chain density. By using either a blot-and-spray or a direct mixing and spraying approach we can produce grids that display clear mixing of the samples, as demonstrated by the dissociation of myosin-S1 from the actin filaments.

Recent studies have suggested that rapid vitrification of grids can minimize the interactions with the air–water interface, which is detrimental to the biological sample (Noble *et al.*, 2018 ▸; D’Imprima *et al.*, 2019 ▸). The methodology reported here is capable of vitrifying grids rapidly and reproducibly, which may alleviate some of the problems associated with interactions at the air–water interface, as well as other problems in conventional blotting systems (Glaeser *et al.*, 2016 ▸; Glaeser, 2018 ▸). Ribosome data collected from sprayed grids suggest a broad distribution of views [Fig. S1(*c*)]. This is consistent with the particles making fewer interactions with the air–water interface; however, a full systematic study of this is beyond the scope of this work.

The grid preparation procedure is still a significant bottleneck in cryo-EM and has plenty of room for improvement both in terms of reproducibility and in the development of TrEM applications. A number of new approaches have emerged which aim to produce more consistent and reproducible high-quality ice, for example Spotiton and VitroJet systems (Dandey *et al.*, 2018 ▸; Ravelli *et al.*, 2019 ▸). Here we report a system that addresses a different problem, that of the rapid mixing and trapping of different conformational states to produce cryo-EM grids sufficient for high-resolution EM structure determination. By using a voltage-assisted spray and a rapid mixing unit we can directly spray onto rapidly plunging EM grids or mix and freeze grids with an ∼10 ms time delay. This integrated apparatus may allow us to open up new opportunities in understanding the mechanisms of different protein systems.

## Supplementary Material

Supporting Movie 1. DOI: 10.1107/S2052252519011345/pw5007sup1.mov

Supporting Movie 2. DOI: 10.1107/S2052252519011345/pw5007sup2.mov

Supporting Movie 3. DOI: 10.1107/S2052252519011345/pw5007sup3.mov

Supporting information. DOI: 10.1107/S2052252519011345/pw5007sup4.pdf
