# Supplementary material for: A cryo-EM grid preparation device for time-resolved structural studies
Source: IUCrJ. 2019 Sep 5;6(Pt 6):1024–31. doi: 10.1107/S2052252519011345 (PMC6830222; doi:10.1107/S2052252519011345)
Supplement: Supplementary file 4 [file m-06-01024-sup4.pdf]

IUCrJ

Volume 6 (2019)

Supporting information for article:

A cryo-EM grid preparation device for time-resolved structural studies

Dimitrios Kontziampasis, David P. Klebl, Matthew G. Iadanza, Charlotte A. Scarff, Florian Kopf, Frank Sobott, Diana C. F. Monteiro, Martin Trebbin, Stephen P. Muench and Howard D. White

Table S1 Data collection statistics for the three reported datasets, Apoferritin, ribosome and PV-TF.

	Apoferritin	Ribosome	PV-TF
Data collection			
Magnification	75000	75000	75000
Voltage (kV)	300	300	300
Electron dose (e-/A2)	91	66	65
Defocus range (um)	-1.5 to -3.4	-0.8 to -2.9	-1.3 to -4
Pixel size (A)	1.07	1.07	1.07
Processing			
Initial number of particles	225,604	47,866	107,121 segments (3316 filaments)
Final number of particles	34,170	34,010	93,468
Symmetry	O	C1	H<br>Twist: -166.33<br>Rise: 27.65 A
Pre-polishing global resolution (FSC = 0.143)	3.9	5.2	8.0
Global resolution (FSC = 0.143)	3.6	4.3	5.6

Table S2 Analysis of the ice quality on the sprayed grids for the apoferritin, ribosome and thin filament data.

	thick ice	thin ice
Apoferritin	51 %	0.6 %
Ribosome	29 %	0.5 %
Thin filaments	20 %	0.5 %
Mean	33 %	0.5 %

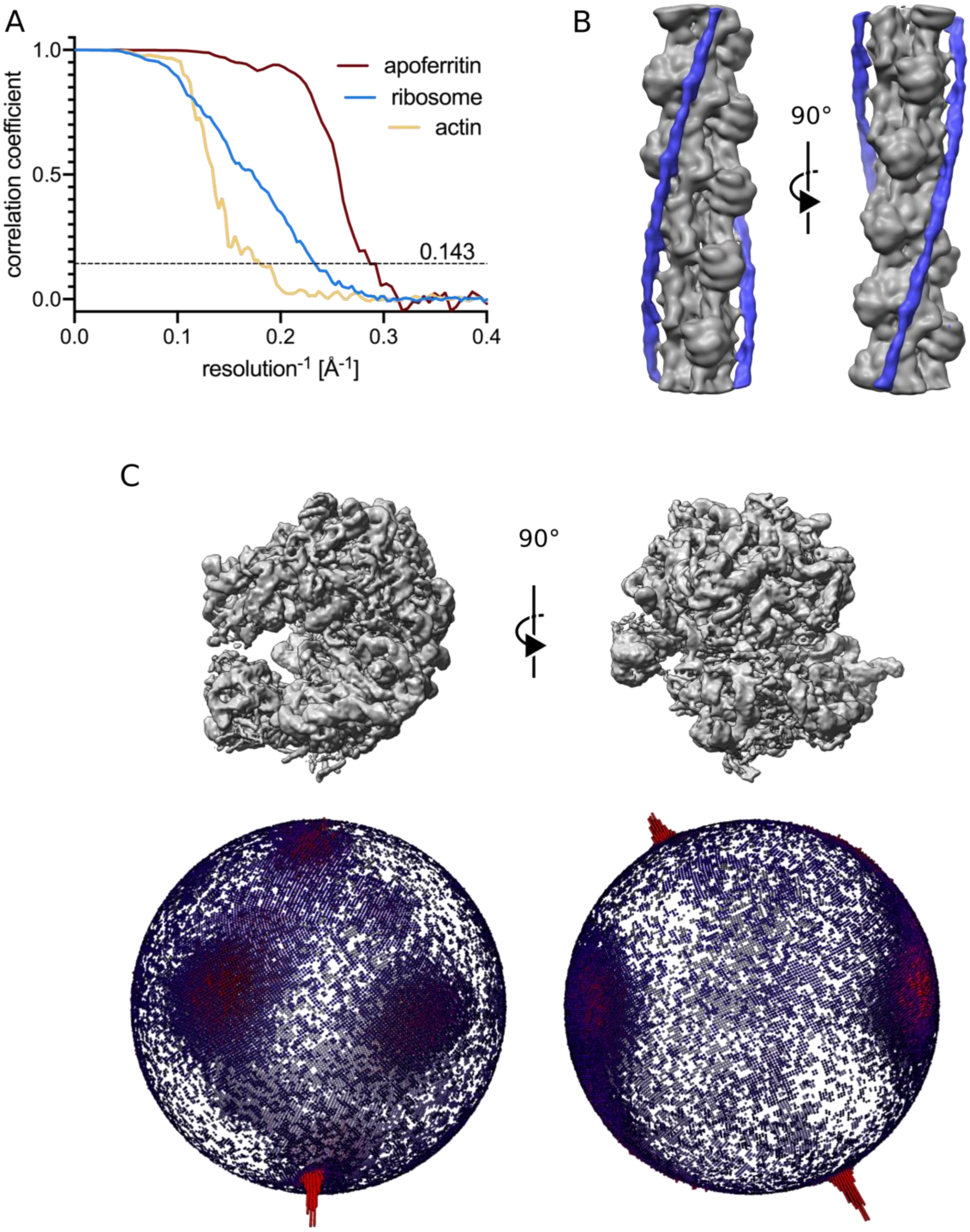

Figure S1 Processing data from sprayed grids. (A) Corrected FSC curves for the final masked maps of apoferritin, actin and ribosome. The dashed line represents the 0.143 cut-off. (B) Tropomyosin-containing sub-class (9496 particles) from the PV-TF dataset at 10.4 Å resolution, the actin backbone is colored in grey and tropomyosin in blue. (C) Spread of angular orientation for the ribosome dataset.

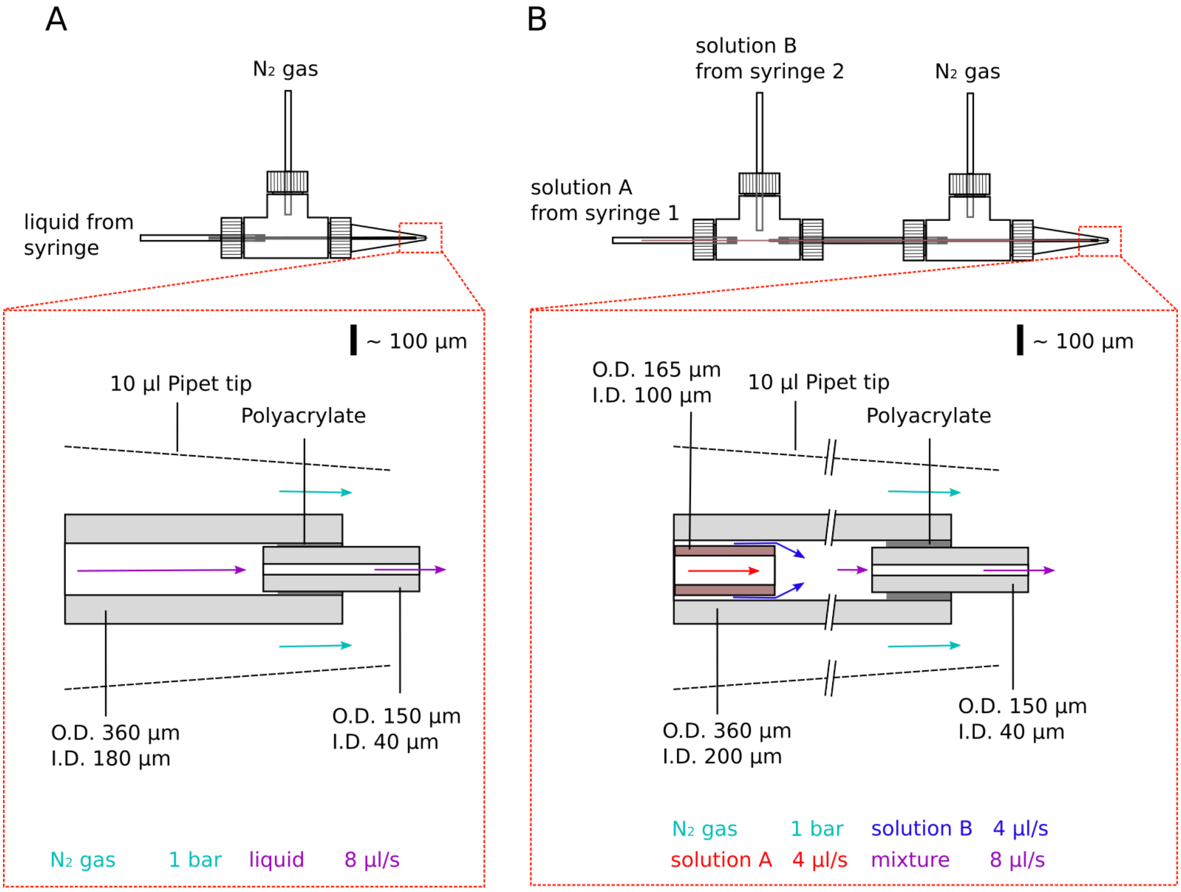

Figure S2 Schematic of the standard spraying unit (A) and the mixing/spraying unit (B) with an overview at the top and magnified view of the spray tip below.

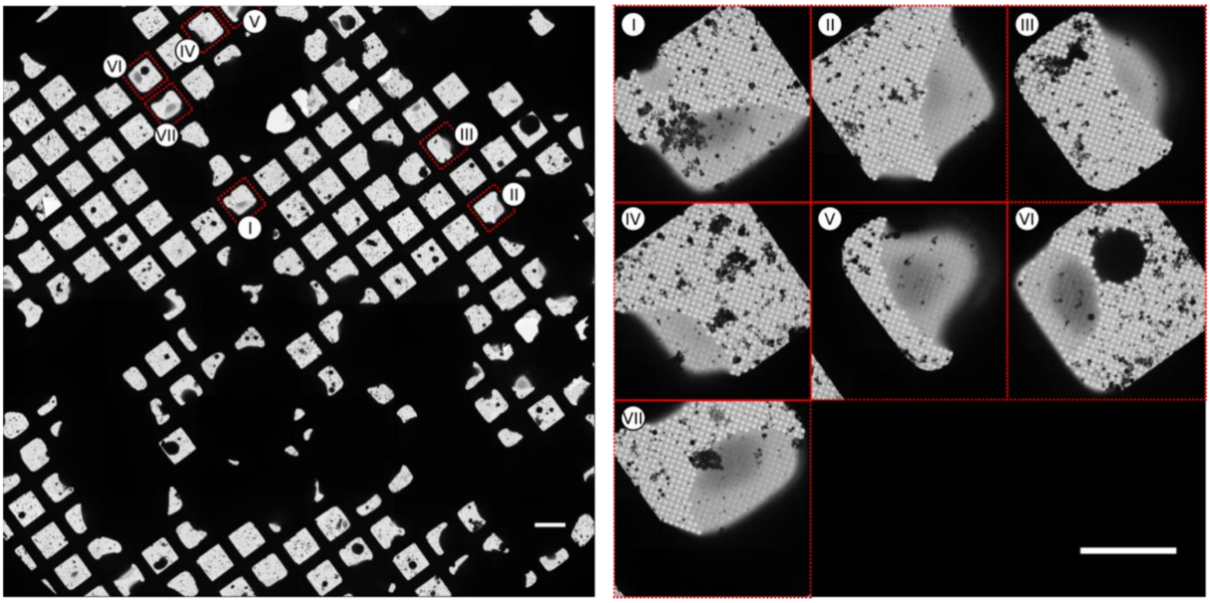

Figure S3 Selection of grid squares for data collection (exemplified for the apoferritin dataset). The image on the left shows a low magnification montage (atlas view) of grid with areas used in data collection highlighted in red (scale bar: 100 µm). The image on the right shows higher magnification micrographs of the selected grid squares with the numbers highlighting the position on the atlas (scale bar: 50 µm).

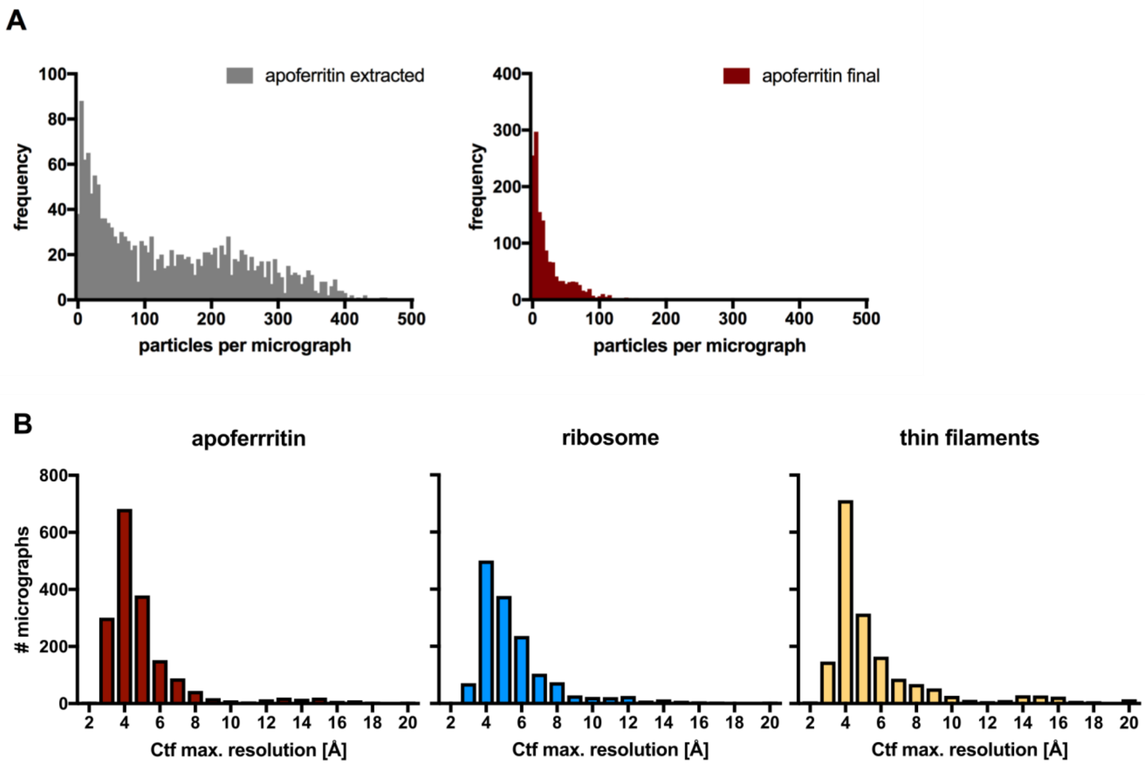

Figure S4 (A) Histograms showing the distribution of particle number per micrograph in the apoferritin dataset. Before any classification, micrographs contributed between 1 and 460 particles to the dataset, after curation of the dataset through 2D classification and selection for particles in high resolution classes, micrographs contributed mainly low particle numbers, between 1 and 175 particles, showing that 'good' particles are spread throughout the dataset. (B) Thon ring analysis for all micrographs in the apoferritin, ribosome and thin filament datasets, estimated by Gctf (Zhang 2016) showing the main peak lying between 3-5Å.

Movie S1 Real time movie of the TrEM setup operating in the basic spray mode to generate grids with no pre-mixing or blotting. Note that the voltmeter is resting upon the high tension voltage module (III in Figure 1) rather than standing at the side.

Movie S2 Real time movie of the TrEM setup operating in 'blot and spray' mode. The setup is the same as in movie S1 with the sprayer as shown in Fig. S2A.

Movie S3 Real time movie of the TrEM setup operating in 'mix and spray' mode with the sprayer design as shown in Fig. S2B.
